# Frontal Lobe Function and Risk of Hip Fracture in Patient With Alzheimer Disease: An Analysis of Linked Data

**DOI:** 10.1097/MD.0000000000001918

**Published:** 2015-11-13

**Authors:** Hyun Woong Roh, Chang Hyung Hong, SooJin Lee, Yunhwan Lee, Kang Soo Lee, Ki Jung Chang, Byoung Hoon Oh, Seong Hye Choi, Seong Yoon Kim, Joung Hwan Back, Young Ki Chung, Ki Young Lim, Jai Sung Noh, Sang Joon Son

**Affiliations:** From the Department of Psychiatry, Ajou University School of Medicine, Suwon (HWR, CHH, KJC, YKC, KYL, JSN, SJS); Health Administration, Department of Management & Administration, Backseok Arts University, Seoul (SJL); Department of Preventive Medicine and Public Health, Ajou University School of Medicine (YL); Institute on Aging, Ajou University Medical Center, Suwon (HWR, CHH, YL, KJC, SJS); Department of Psychiatry, CHA University College of Medicine, Gangnam Medical Center and CHAUM Life Center (KSL); Department of Psychiatry and Institute of Behavioral Science in Medicine, Yonsei University College of Medicine, Seoul (BHO); Department of Neurology, Inha University School of Medicine, Incheon (SHC); Department of Psychiatry, Asan Medical Center, University of Ulsan College of Medicine, Seoul (SYK); and Health Insurance Police Research Institute, National Health Insurance Service, Seoul, Republic of Korea (JHB).

## Abstract

To determine the association between frontal lobe function and risk of hip fracture in patients with Alzheimer disease (AD).

Retrospective cohort study using multicenter hospital-based dementia registry and national health insurance claim data was done. Participants who had available data of neuropsychological test, national health insurance claim, and other covariates were included. A total of 1660 patients with AD were included based on Stroop Test results. A total of 1563 patients with AD were included based on the Controlled Oral Word Association Test (COWAT) results. Hip fracture was measured by validated identification criteria using national health insurance claim data. Frontal lobe function was measured by Stroop Test and COWAT at baseline.

After adjusting for potential covariates, including cognitive function in other domains (language, verbal and nonverbal memory, and attention), the Cox proportional hazard regression analysis revealed that risk of a hip fracture was decreased with a hazard ratio (HR) of 0.98 per one point of increase in the Stroop Test (adjusted HR = 0.98, 95% confidence interval [CI]: 0.97–1.00) and 0.93 per one point increase in COWAT (adjusted HR = 0.93, 95% CI: 0.88–0.99).

The risk of hip fracture in AD patients was associated with baseline frontal lobe function. The result of this research presents evidence of association between frontal lobe function and risk of hip fracture in patients with AD.

## INTRODUCTION

Alzheimer disease (AD) and hip fracture are predominantly occurring diseases in the elderly (≥65 years). Their high morbidity and mortality give heavy burdens to both patients and their caregivers. A few prior studies have suggested that the elderly with AD have higher risk of hip fracture compared to cognitively intact elderly.^1–7^ However, some of these research studies were based on cross-sectional design that it was difficult to draw a conclusion on their etiologic association.^3,5,8^ Although some research studies were based on longitudinal design, only limited characteristics of AD such as Mini-mental state examination (MMSE) score or insufficient numbers of subjects were used. Therefore, it was difficult to find out specifically what factors of AD patients affected the occurrence of hip fracture.^2,6,7^

The main objective of the present study was to assess the longitudinal association of frontal lobe function and risk of hip fracture in AD patients. A few prior research studies have reported that frontal lobe function is associated with increased fall risk in cognitively intact elderly.^9–11^ We placed a finer focus on research subjects by involving exclusively patient with AD. Main outcome was not focused simply on falls, but more specifically on hip fracture that exerts great effect on morbidity and mortality. Such a specific research setting could secure a higher clinical significance. We hypothesized that impaired frontal lobe function would increase the risk of hip fracture in AD patients. To determine the association between frontal lobe function and the risk of hip fracture in AD patients, we analyzed longitudinally linked data of Clinical Research Center for Dementia of South Korea (CREDOS) which has a wide range of baseline information including standardized neuropsychological test results and National Health Insurance (NHI) Claims Database of the Health Insurance Review & Assessment Service (HIRA).

## METHODS

### Participants

The study population comprised CREDOS study participants aged 65 years and above who were diagnosed with AD. The CREDOS study registered on ClinicalTrials.gov (identifier: NCT01198093) recruited participants from university-affiliated hospitals who were diagnosed with normal cognition, subjective memory impairment, mild cognitive impairment, vascular cognitive impairment, subcortical ischemic vascular dementia, or AD by neurologist or psychiatrist. In the CREDOS study, we used the diagnostic criteria for probable AD issued by the National Institute of Neurological and Communicative Disorder and Stroke-Alzheimer's Disease and Related Disorder Association (NINCDS-ADRDA).^12^ A more detailed description of CREDOS study has been published previously.^13,14^ To identify the occurrence of hip fracture, we used the NHI Claims Database of the HIRA. South Korea has a universal national health coverage system that covers approximately 98% of overall South Korean population.^15^ A more detailed description of HIRA database is published previously.^16^

Of CREDOS study participants from January 1, 2008 to August 31, 2012, we retrospectively analyzed the NHI claim data from the time of participant's CREDOS study enrollment up to August 31, 2012 or the time of event (hip fracture or death). Participants who had available data of neuropsychological test, NHI claim, and other covariates were included. We excluded those who met following criteria: (1) history of hip fracture within 6 months at the time of CREDOS study enrollment; (2) history of significant hearing or visual impairment rendering participation in the interview difficult; (3) history of following neurologic disorder (brain tumor, intracranial hemorrhage, subarachnoid hemorrhage, epilepsy, hydrocephalus, encephalitis, and metabolic encephalopathy) or other neurologic conditions that could interfere with the study; (4) history of following psychiatric disorder (mental retardation, schizophrenia, and bipolar disorder) or other psychiatric conditions that could interfere with the study; (5) history of using psychoactive substances other than alcohol; (6) history of following physical illnesses or disorders (cancer, renal failure, hepatic failure, uncontrolled diabetes, severe asthma or chronic obstructive pulmonary disease, syphilis and abnormal thyroid function test) or other physical conditions that could interfere with the study.

### Standard Protocol Approvals, Registrations, and Patient Consents

CREDOS study was approved by the institutional review board of the participating centers. All participants signed informed written consents. This study was approved by the institutional review boards at the clinical sites. We were provided with permission to use the NHI claim database by the HIRA and Ministry of Health & Welfare.

### Primary Measurements

Cognitive function was assessed at baseline with a standardized neuropsychological battery of the Seoul Neuropsychological Screening Battery-Dementia Version (SNSB-D).^17^ The SNSB-D included tests of language, verbal/nonverbal memory, attention, and frontal lobe function domains. These domains were assessed using the following tests: the Korean short version of the Boston Naming Test for language, the Digit Span Test-backward for attention function, the Rey Complex Figure Test-delayed recall for visuospatial memory, the Seoul Verbal Learning Test-delayed recall for verbal memory, and Stroop Test and Controlled Oral Word Association Test (COWAT) for frontal lobe function. Stroop Test or COWAT scores below the one standard deviation (16th percentile) of the age, gender, and education specific norm were classified as impaired in this study based on SNSB-D reference standards.^17^

Occurrence of hip fracture was assessed by validated identification criteria for cases of hip fracture using NHI Claims Database.^18^ The criteria to identify hip fracture using a diagnostic code, a procedure code, the type, and number of medical service usages were developed via discussions among orthopedic and epidemiologic experts. The criteria were validated by using a hip fracture cohort. Fracture due to car accident was not covered by NHI but by automobile insurance system. Therefore, we did not conduct other exclusion process.

### Covariates

Possible covariates included baseline age, gender, and education years. Comorbidity was assessed using Charlson Comorbidity Index.^19–21^ Body mass index (BMI) was calculated from direct height and weight measurements and categorized into 4 groups reflecting World Health Organization Asian standard.^22^ Parkinsonian gait was determined after physical examinations by neurologists or psychiatrists based on the presence of decreased arm swing, stooped posture, short step gait, festination, shuffling, and pivot turning. Depressive symptoms were assessed using the Korean version of 15-item Geriatric Depression Scale (GDS-15).^23^

### Statistical Analyses

Categorical variables were compared using Chi-square tests. Continuous variables were compared using independent Student *t* tests between the group with frontal lobe function impairment and the group without frontal lobe function impairment. The incidence rate of hip-fracture was calculated for the subjects at the end of follow-up. We used Kaplan–Meier survival curves to plot the survival curve for incident hip fracture by the presence of frontal lobe impairment. Cox proportional hazards regression was created to estimate the hazard ratios (HRs) and 95% confidence intervals (CIs) of hip-fracture after adjusting demographic factors, comorbidities, BMI, parkinsonian gait, GDS-15 score, and other cognitive function score. All statistical analyses were performed using Statistical Analysis System (SAS) version 9.3 (SAS Institute, Inc., Cary, NC). Statistical significance was considered when *P*-value was less than 0.05.

## RESULTS

Baseline characteristics of participants are summarized in Table 1. Based on Stroop Test and COWAT, 1660 and 1563 patients with AD were included, respectively. One thousand four hundred fifty-nine participants who completed both tests were analyzed twice in each group, once as the Stroop Test group and once as the COWAT group.

**TABLE 1:**
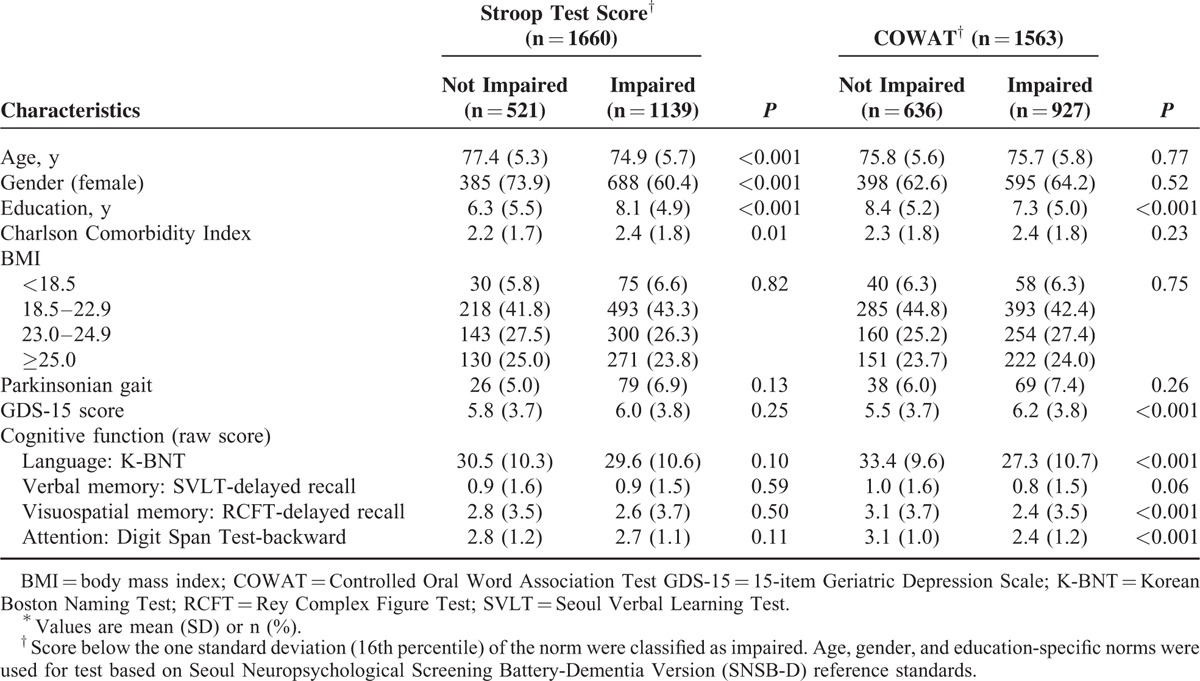
Baseline Characteristics by Frontal Lobe Function Among Participants^∗^

**FIGURE 1:**
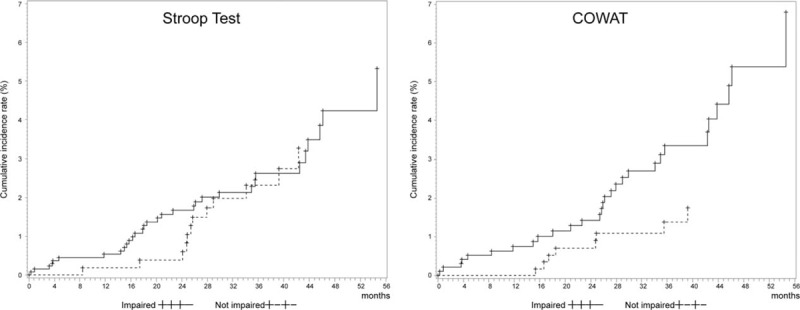
Kaplan–Meier survival function of time to hip fracture by frontal lobe function test^a^. ^a^Score below the one standard deviation (16th percentile) of the norm were classified as impaired. Age, gender, and education-specific norms were used for test based on Seoul Neuropsychological Screening Battery-Dementia Version (SNSB-D) reference standards. COWAT = Controlled Oral Word Association Test.

Based on Stroop Test result, the longest follow-up duration was 54.7 months. Over the follow-up, 44 (2.7%) participants developed incidents of hip fracture. AD patients who had impaired Stroop Test at baseline were more likely to develop hip fracture (n = 32, 2.8%) compared to those who did not have impaired Stroop Test at baseline (n = 12, 2.3%, Fig. 1). In the unadjusted Cox proportional hazard models, the risk of hip fracture was decreased with HR of 0.98 per one point increase in Stroop Test. Direction and strength of the association between the Stroop Test and risk of hip fracture in patient with AD remained even after adjusting for potential confounders including cognitive function in other domains (language, verbal and nonverbal memory, and attention). In the adjusted Cox proportional hazard models, the risk of hip fracture was decreased with HR of 0.98 per one point increase in Stroop Test (Table 2).

**TABLE 2:**
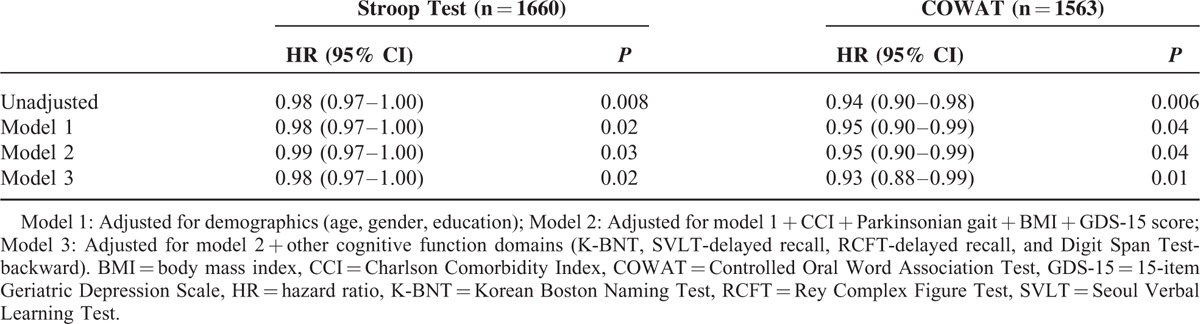
Cox Proportional Hazard Ratios for Time to Hip Fracture by One Point Increase of Frontal Lobe Function Test

Based on COWAT result, the longest follow-up duration was 54.7 months. Over the follow-up, 39 (2.5%) participants developed incidents of hip fracture. AD patients who had impaired COWAT at baseline were more likely to develop hip fracture (n = 31, 3.3%) compared to those who did not have impaired COWAT at baseline (n = 8, 1.3%, Fig. 1). In the unadjusted Cox proportional hazard models, the risk of hip fracture was decreased with HR of 0.94 per one point increase in COWAT. Direction and strength of the association between the COWAT and risk of hip fracture in patient with AD remained even after adjusting for potential confounders including cognitive function in other domains (language, verbal and nonverbal memory, and attention). In the adjusted Cox proportional hazard models, the risk of hip fracture was decreased with HR of 0.93 per one point increase in COWAT (Table 2).

## DISCUSSION

The primary purpose of this study was to investigate the association between frontal lobe function and risk of hip fracture in patient with AD. Our study demonstrated that the risk of hip fracture in AD patients was associated with baseline frontal lobe function after adjusting for age, gender, and education. The association was still significant after adjusting for potential confounders, including comorbidities, BMI, parkinsonian gait, depressive symptoms, and other cognitive function domains.

The possible interpretation linking frontal lobe function impairment to increased risk of hip fracture in AD patients could be considered in several ways. First, gait disturbance and increased risk of fall, which could be associated with frontal lobe function impairment, might be a significant cause of increased risk for hip fractures. Prior research has revealed that AD patients show gait disturbance accompanied by slower walking velocity, decreased step length, and increased step length variation, which are all associated with an increased risk of fall.^24–26^ Meanwhile, some researchers reported that such gait disturbance of AD patients was associated with their frontal lobe function impairment.^27^ Recent review articles, and a few prospective studies about neuropsychological mechanisms of falls in elderly, also support similar ideas.^10,28,29^ In general, aging causes cognitive shifting from unconscious to increasingly conscious information processing, resulting in greater reliance on frontal lobe function.^30^ Consequently, seemingly easy and automatic tasks such as walking could become difficult and require greater conscious control.^31^ Such a change, however, is not a sufficiently stable process. It is accompanied by problems in motor performance increasing the risk of injury in the elderly.^31–33^ According to this concept, AD patients with frontal lobe function impairment are anticipated to show poor compensation for their cognitive shifting. They may show severe gait disturbance, the so-called frontal gait disorder, and are at a higher risk of falls.^34,35^ In the present research, however, gait measurement was insufficient and falls were not included as covariates in regression models. So such an association could not be clearly identified. Future research requires direct information about gait and history of falls and, based on them, an approach should be made to the relationship between frontal lobe function, gait disturbance, history of falls, and risk of hip fractures in AD patients. Second, general intelligence factor (G factor) associated with frontal lobe function may have contributed to the risk of hip fracture in AD patients. The concept of G factor is based on the presence of one dominant general intelligence factor that influences performance on narrow task-specific cognitive abilities.^36^ Recent researches have shown a close association between frontal lobe function and general intelligence.^37^ In the present research, the COWAT impairment group, which gave more significant results, also exhibited significantly lower scores in other cognitive domains, indicating the possibility of the contribution of G factor. To make an approach to such a concern, the analysis in our research involved an adjustment of other cognitive function domains. Yet, this can hardly be considered sufficient for addressing the independent effects of frontal lobe function and G factor. Follow-up researches would need a statistical approach, such as the structural equation model, that enables a more accurate assessment of independent effects. Third, frontal lobe function impairment could be a general indicator of dementia progression accompanying comorbidity, sarcopenia, and depression. These accompanying features were known to have influence on hip fracture development.^38–40^ However, our results were robust to adjustment for comorbidity score, parkinsonian gait, BMI, and depression. Fourth, restless or uncoordinated behavior associated with frontal lobe function impairment could have increased the risk of hip fracture. In previous researches, AD patients with frontal lobe function impairment have shown more behavioral problems such as agitation or aberrant motor behavior. Such problems could expose patients to more risks of hip fracture.^41–43^ Although Stroop Test and COWAT are known to be partly related to the extent of impulsivity and aggressiveness, the implications of these findings for hip fracture predictions are unclear.^43,44^

An important implication of this study is that various factors that should be considered in future research have been confirmed. Of them, the authors strongly felt the necessity of a more thorough executive function and gait performance test. An alternative for such a test could be the dual task test used in recent researches for measuring executive function on gait.^45^ In addition, the authors also strongly felt the need for a direct measurement of bone mass and sufficient information about fall history. A few prior research studies have suggested that AD could increase the risk of fracture by increasing the risk of falls and also by reducing the bone mineral density, probably through the degeneration in the hypothalamus.^46,47^ Clinical, neuropathological, and neuroimaging data together exhibited that the hypothalamus is affected in AD and undergoes neuronal loss, profound plaque and tangle formation, and overall atrophy.^48–51^ Also, atrophy of the hypothalamus and loss of hypothalamic neurons were associated with bone mineral density in AD patients.^46^ Therefore, the bone mineral density and hypothalamic degeneration should be considered as confounding factors in future research to provide etiologic evidence. Finally, the authors also strongly felt the necessity of a more direct measurement, rather than the indirect indices, of sarcopenia and frailty.

The possibility that AD patients at increased risk for hip fracture could be identified by a frontal lobe function test is another implication of this study that has been confirmed. Much research has been done on fall prevention for the elderly population. So, what is more important now is to whom the intervention should be applied to.^52^ In this regard, the present research presents further evidence-based information.

Our study has several strengths, including a longitudinal design with a long follow-up period up to 54.7 months and a relatively large sample of patient with AD. Also, diagnostic classification of our study was carried out directly by a neurologist or a psychiatrist who had abundant clinical experiences. Finally, corrections were also made for diverse potential covariates, including those that have previously been shown to be related to AD and hip fracture.

There are several limitations that should be taken into consideration when interpreting our study results. First, our study cannot provide a sufficient explanation regarding the various effects shown by the 2 different frontal lobe function tests. The Stroop Test is known to be associated with the dorsolateral prefrontal cortex and orbitofrontal cortex.^53,54^ The orbitofrontal cortex is known to be associated with sympathetico-vagal balance control through the central autonomic network.^55^ Also, recent research have suggested that sympathetic nervous output from the ventral hypothalamus directly regulates bone remodeling through activation of beta-2 adreno-receptors on the osteoblasts, resulting in reduced bone formation.^56^ These autonomous nervous system changes could be associated with increased risk of osteoporosis and hip fracture in AD patients. Whereas COWAT is known to reflect frontal lobe function, no sufficient consensus has yet been developed as to the specific interpretation or neuroanatomical correlates.^57^ Taking such a difference into consideration, we analyzed the Stroop Test and COWAT individually, and found that the Stroop Test was of borderline significance level, while the COWAT was significant. We could presume the reason for such a difference is that the 2 tests exert different effects on general intelligence, autonomic instability, neuroanatomical change, etc. However, since we could not conduct an evaluation to verify such a presumption, we cannot give a sufficient explanation for the difference. Though this was a major limitation of our study, it could be a point of special interest for follow-up researches. Second, there could be selection bias in our study, which is probably caused by little extensive and broad exclusion criteria, such as psychological history, neurologic or physical illnesses, and unperformed Stroop Test or COWAT, for those participants who were unable to complete both tests. Also, more young patients among impaired Stroop Test group indicate a possibility that there could be a selection bias which is probably caused by higher risk of premature death in impaired group. Third, BMI is an indirect and insufficient measure of one's nutritional status or sarcopenia. For instance, an elderly person with sarcopenic obesity is not distinguishable from a healthy muscular elderly person by BMI alone. Future work is greatly needed to attain a more specific measure, such as serum albumin concentration or lower limb strength. Fourth, score below the one standard deviation is not enough to be sure these patients have a definite clinical frontal lobe function deficit. However, in Stroop test group, 20% of subjects who developed hip fracture (n = 9) exhibited baseline standard deviation score between −1.5 and −1.0 of the norm, indicating the possibility of high risk of hip fracture in mild to moderate impairment of Stoop Test group. This could be associated with high-level activity and low-level dependency of mild to moderate AD patients, but our study cannot suggest sufficient explanation about this issue. Finally, no approach was made to frailty or osteoporosis or the use of psychotropic medication that were shown by preceding researches to be associated with increased risk of hip fracture in AD patients.^31,58–61^ In particular, medications that could influence the bone mineral density, such as vitamin D, bisphosphonates, antiepileptic drugs, selective serotonin reuptake inhibitor and proton pump inhibitor should be considered as important confounding factors in future research.

Through some preceding researches, it has been suggested that AD patients have relatively high risk of hip fracture.^1–8^ Yet the mechanism still remains unclear. No approach has been made to access respective involvement of different cognitive function domains. To our knowledge, the result of this research presents the first evidence of association between frontal lobe function and risk of hip fracture in patient with AD.
